# Transient RBBB and Temporary LBBB Following Transcatheter Aortic Valve Replacement

**DOI:** 10.1016/j.jaccas.2025.105741

**Published:** 2025-10-18

**Authors:** Jiayu Liang, Suxin Luo, Bi Huang

**Affiliations:** a Department of Cardiology, The First Affiliated Hospital of Chongqing Medical University, Chongqing, China; b Department of Cardiology, Shanghai Institute of Cardiovascular Diseases, Zhongshan Hospital, Fudan University, Shanghai, China

**Keywords:** left bundle branch block, right bundle branch block, transcatheter aortic valve replacement

## Abstract

**Background:**

Post-transcatheter aortic valve replacement (TAVR) conduction disturbances typically manifest as left bundle branch block (LBBB). Sequential right bundle branch block (RBBB) followed by LBBB is extremely rare.

**Case Summary:**

An 82-year-old female developed transient RBBB 1 hour after TAVR, followed by persistent LBBB 1 day later. Both conduction abnormalities resolved at the 1-month follow-up.

**Conclusions:**

New-onset RBBB may herald subsequent LBBB, warranting extended monitoring periods.

**Take-Home Messages:**

RBBB is uncommon after TAVR, but it should not be underestimated. In patients with RBBB following the procedure, prolonging the duration of temporary pacemaker implantation may be a prudent and clinically reasonable strategy.

## Graphical Abstract

**Figure:**
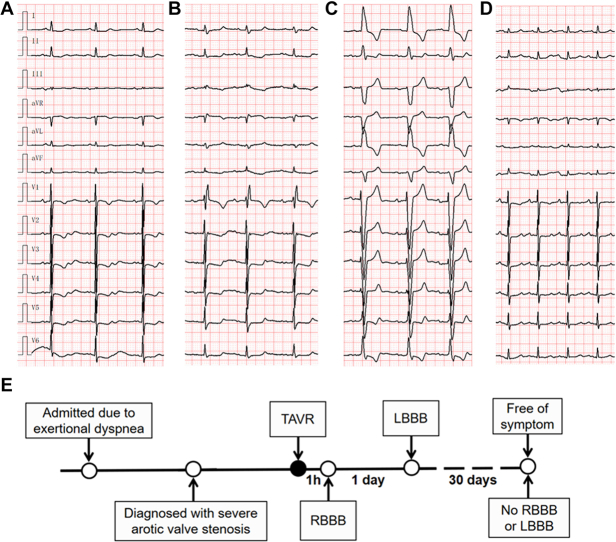

An 82-year-old woman was admitted due to exertional dyspnea. Transthoracic echocardiography revealed the aortic valve area was 0.4 cm^2^. The patient successfully underwent transcatheter aortic valve replacement (TAVR) and had a self-expanding 23.5 × 46-mm Venus A-Valve implanted. The patient was implanted with a temporary pacemaker during the procedure, which was retained post TAVR. The electrocardiogram (ECG) before TAVR is shown in Figure 1A. One hour after the TAVR procedure, the ECG showed complete right bundle branch block (RBBB) (Figure 1B).

**Take-Home Messages**

• Right bundle branch block is uncommon after transcatheter aortic valve replacement, but it should not be underestimated.
• In patients with right bundle branch block following the procedure, prolonging the duration of temporary pacemaker implantation may be a prudent and clinically reasonable strategy.

**Figure 1:**
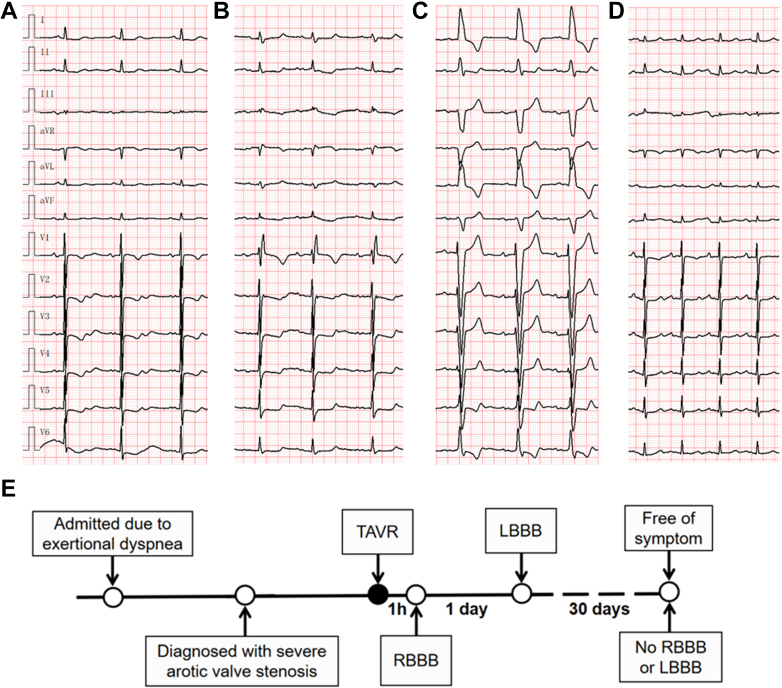
Electrocardiographic Changes Before and After Transcatheter Aortic Valve Replacement (A) ECG showed no atrioventricular block before TAVR; (B) ECG showed RBBB after TAVR; (C) RBBB changed from RBBB to LBBB 1 day after TAVR; (D) atrioventricular block disappeared during follow up. (E) Timeline of the patient's diagnostic and treatment. LBBB = left bundle branch block; RBBB = right bundle branch block; TAVR = transcatheter aortic valve replacement.

Which of the following is correct regarding the clinical significance of RBBB post TAVR?

(A) Does not increase the risk of permanent pacemaker implantation (PPI).
(B) Not related to the damage of the cardiac conduction system.
(C) Prolonged monitoring with a temporary pacemaker may be necessary.
(D) Requires immediate treatment with PPI.

The correct answer is C.

For this patient, 1 day after TAVR, ECG displayed complete left bundle branch block (LBBB) (Figure 1C). During the first 24 hours after TAVR, while under the protection of a temporary pacemaker, the patient reported no discomfort. Subsequent Holter and ECG monitoring indicated a persistent LBBB during hospitalization. At the 30-day follow-up after discharge, ECG showed resolution of the LBBB (Figure 1D). The patient's entire treatment course and the timeline of electrocardiographic changes are presented in Figure 1E.

Preexisting RBBB is a well-established risk factor for PPI following TAVR.^1^ However, the prognostic significance of newly developed RBBB after TAVR is poorly studied. Tan et al^2^ explored 1992 patients undergoing TAVR and found post-TAVR RBBB was significantly associated with increased risk of PPI.

The case we report, involving RBBB followed by LBBB after TAVR, is extremely rare. The exact mechanism remains unclear, but there are some possible interpretations. The His bundle serves as the common origin for the left and right bundle branches. Damage to the bundle branches can manifest as selective or sequential bundle branch block. The progression from RBBB to LBBB post TAVR typically signifies a sequential impairment of the conduction system, wherein the right bundle branch is initially affected, followed by involvement of the left bundle branch or its fascicles. In addition to anatomical factors, procedure-related aspects of TAVR, such as mechanical compression by the valve, valve type and size, and localized inflammation or edema following valve implantation, may all contribute to the development of conduction block.

Current expert consensus recommends that patients who develop new-onset LBBB after TAVR should be monitored for at least 24 hours following temporary pacemaker implantation.^3^ In our case, the patient developed RBBB post TAVR, which progressed to LBBB the following day. During this period, the patient was under the protection of a temporary pacemaker. If the patient had not been supported by a temporary pacemaker, and LBBB had occurred without resolution of RBBB, this could have resulted in complete atrioventricular block, potentially leading to syncope or even sudden cardiac death. Unfortunately, there is a lack of electrocardiographic data capturing the transition from RBBB to LBBB and the accurate timing of this transition. Therefore, new-onset RBBB may herald subsequent LBBB, warranting extended monitoring periods.

## Funding Support and Author Disclosures

The authors have reported that they have no relationships relevant to the contents of this paper to disclose.
